# Whole genome sequencing reveals population diversity and variation in HIV-1 specific host genes

**DOI:** 10.3389/fgene.2023.1290624

**Published:** 2023-12-20

**Authors:** Prisca K. Thami, Wonderful T. Choga, Collet Dandara, Stephen J. O’Brien, Myron Essex, Simani Gaseitsiwe, Emile R. Chimusa

**Affiliations:** ^1^ Division of Human Genetics, Department of Pathology, University of Cape Town, Cape Town, South Africa; ^2^ Botswana Harvard AIDS Institute Partnership, Gaborone, Botswana; ^3^ Institute of Infectious Disease and Molecular Medicine, University of Cape Town, Cape Town, South Africa; ^4^ UCT/SAMRC Platform for Pharmacogenomics Research and Translation (PREMED) Unit, South African Medical Research Council, Cape Town, South Africa; ^5^ Laboratory of Genomics Diversity, Center for Computer Technologies, ITMO University, St. Petersburg, Russia; ^6^ Guy Harvey Oceanographic Center Halmos College of Arts and Sciences, Nova Southeastern University, Fort Lauderdale, FL, United States; ^7^ Department of Immunology and Infectious Diseases, Harvard T. H. Chan School of Public Health AIDS Initiative, Harvard T. H. Chan School of Public Health, Boston, MA, United States; ^8^ Department of Applied Sciences, Faculty of Health and Life Sciences, Northumbria University, Newcastle, United Kingdom

**Keywords:** genome variation, population genetics, human immunodeficiency virus (HIV-1), genomics, functional prediction, Botswana, Africa

## Abstract

HIV infection continues to be a major global public health issue. The population heterogeneity in susceptibility or resistance to HIV-1 and progression upon infection is attributable to, among other factors, host genetic variation. Therefore, identifying population-specific variation and genetic modifiers of HIV infectivity can catapult the invention of effective strategies against HIV-1 in African populations. Here, we investigated whole genome sequences of 390 unrelated HIV-positive and -negative individuals from Botswana. We report 27.7 million single nucleotide variations (SNVs) in the complete genomes of Botswana nationals, of which 2.8 million were missing in public databases. Our population structure analysis revealed a largely homogenous structure in the Botswana population. Admixture analysis showed elevated components shared between the Botswana population and the Niger-Congo (65.9%), Khoe-San (32.9%), and Europeans (1.1%) ancestries in the population of Botswana. Statistical significance of the mutational burden of deleterious and loss-of-function variants per gene against a null model was estimated. The most deleterious variants were enriched in five genes: *ACTRT2* (the Actin Related Protein T2), *HOXD12* (homeobox D12), *ABCB5* (ATP binding cassette subfamily B member 5), *ATP8B4* (ATPase phospholipid transporting 8B4) and *ABCC12* (ATP Binding Cassette Subfamily C Member 12). These genes are enriched in the glycolysis and gluconeogenesis (*p* < 2.84e-6) pathways and therefore, may contribute to the emerging field of immunometabolism in which therapy against HIV-1 infection is being evaluated. Published transcriptomic evidence supports the role of the glycolysis/gluconeogenesis pathways in the regulation of susceptibility to HIV, and that cumulative effects of genetic modifiers in glycolysis/gluconeogenesis pathways may potentially have effects on the expression and clinical variability of HIV-1. Identified genes and pathways provide novel avenues for other interventions, with the potential for informing the design of new therapeutics.

## Introduction

The study of human genomes has revolutionized our understanding of human biology, population diversity, and disease susceptibility (Collins and Fink, 1995; International Human Genome Sequencing Consortium, 2004). Despite Africa being the cradle of humanity (Berger et al., 2015; Hublin et al., 2017), the vast majority of genetic studies have been conducted on people of European ancestry, while only 2% have been carried out on populations of African descent (Beltrame et al., 2016; McGuire et al., 2020; Wonkam and Adeyemo, 2023). Moreover, the human reference genome does not entirely capture variants found in African genomes (Sherman et al., 2019). This underrepresentation has led to a significant disparity in the understanding of the genetic architecture of African populations, potentially biasing our understanding of the genetic etiology of diseases and limiting therapeutic development (Sirugo et al., 2019). Studying the human genomes of Africans is imperative to confront this disparity. By studying genetic variation in African populations, we can gain insights into population history, disease susceptibility, and drug response, which ultimately will lead to better diagnoses, treatments, and a better overall healthcare system for people of African ancestry (Bentley et al., 2017; Wonkam et al., 2022).

There is a disproportionate burden of infectious diseases in Africa and HIV is one of the most prevalent in the region (Nkengasong and Tessema, 2020; Niohuru, 2023). Southern and eastern Africa carry the highest prevalence of human immunodeficiency virus (HIV) infection globally. Botswana is the third most affected country in Southern Africa, followed by eSwatini and South Africa which are in first and second positions, respectively (UNAIDS, 2019). The country is affected predominantly by HIV-1C. The HIV epidemic became severe in Botswana by the late 1990s at a prevalence of 30%–40% in pregnant women (Essex, 1999), reducing to the current 20.8% (Statistics Botswana, 2022) due to the rapid scale-up of anti-HIV drugs that has led to a sharp decline in morbidity and mortality (Farahani et al., 2014; Escudero et al., 2019; Statistics Botswana, 2022). Nonetheless, Botswana remains one of the most affected countries globally due to the high baseline HIV prevalence.

Here, we investigate genetic mutation burden and assess population genetic diversity in an HIV-1 cohort from Botswana.

Leveraging whole genome sequencing (WGS) of Botswana and existing genome databases, this work aimed to 1) unravel genetic variation and substructure within an HIV-1 cohort of Botswana, 2) assess diversity between the Botswana population and other global populations, and 3) study variation in HIV-1 specific host genes. Prioritizing variants in medical genetics entails distinguishing background benign variants from pathogenic variants that can lead to disease phenotypes (Conrad et al., 2010; Torkamani et al., 2011). Therefore, we perform *in silico* functional annotation using many tools and aggregate the classifications to predict pathogenicity of variants (Bope et al., 2019; Chimusa et al., 2022). Notably, our identified deleterious and loss-of-function variants are enriched in pathways associated with relevant pathophysiological mechanisms, including some that are already therapeutic targets. This study fills an important gap in knowledge by using a WGS approach focusing on deleterious variants important in HIV-1 status.

## Materials and methods

### Ethical approval

This study is part of a bigger protocol titled “Host Genetics of HIV-1 Subtype C Infection, Progression and Treatment in Africa/GWAS on determinants of HIV-1 Subtype C Infection” conducted by Botswana Harvard AIDS Institute Partnership. Ethics approval was obtained according to The Declaration of Helsinki. All participants provided written informed consent. Institutional Review Board (IRB) approval was obtained for these samples from Botswana Ministry of Health and Wellness—Health Research Development Committee (HRDC) & Harvard School of Public Health IRB (reference number: HPDME 13/18/1) and the University of Cape Town—Human Research Ethics Committee (HREC reference number: 316/2019).

### Selection of study participants and data acquisition

#### Botswana population

This is a retrospective study that used samples from previous studies conducted at Botswana Harvard AIDS Institute Partnership between 2001 and 2007. Of the 390 participants, 265 were HIV-1 positive and 125 were HIV-1 negative (Supplementary Table S1A). The participants were recruited from four locations within the southern region of Botswana (Mochudi, Molepolole, Lobatse and Gaborone; Supplementary Figure S1). The HIV-1 positive participants were previously part of the Mashi study (Shapiro et al., 2006; Thior et al., 2006), while HIV-1 negative participants were previously part of the Tshedimoso study (Novitsky et al., 2008).

DNA was extracted from buffy coat using Qiagen DNA isolation kit following manufacturer’s instructions. DNA concentration was quantified using Nanodrop^®^ 1000 (Thermo Scientific, United States). Whole genome sequences of 394 Botswana nationals were generated using paired end libraries on Illumina HiSeq 2000 sequencer at BGI (Cambridge, MA, US).

Quality assessment was performed on paired-end WGS (minimum of 30X depth) in FASTQ format (Cock et al., 2010) using FastQC (Van Der Auwera et al., 2014). Low-quality sequence bases and adapters were trimmed using Trimmomatic with default parameters (Bolger et al., 2014). The sequencing reads were aligned to the GRCh38 human reference genome using Burrows-Wheeler Aligner (BWA-MEM) (Li et al., 2008; Li and Durbin, 2009) and post-alignment quality control including adding of read groups, marking duplicates, fix mating and recalibration of base quality scores was performed using Picard tools, SAMtools (Li, 2011) and Genome Analysis Toolkit (GATK) (McKenna et al., 2010). Four samples (HIV-1 positive females) were excluded due to poor quality of sequences, the remaining dataset had 390 individuals. We have run FastQC on all final BAM files prior the variant calling, then we aggregated the results from FastQC into a single report by using MultiQC (Ewels et al., 2016). All the remaining sequences passed quality control.

We performed population joint calling (Nielsen et al., 2011; Pfeifer, 2017) using two different population joint calling methods to leverage the quality and accuracy of our results: GATK’s HaplotypeCaller (McKenna et al., 2010; DePristo et al., 2011) and BCFtools (Li, 2011). The variant call format (VCF) dataset was filtered using VCFTOOLS (Danecek et al., 2011), GATK’s Variant Quality Score Recalibration and BCFtools. The specific filtering parameters employed for both call-sets have been detailed in Supplementary Material. Downstream analyses were performed with GATK call-set and BCFtools call-set used as a validation set.

#### 1000 genomes project and African genome variation project data

We assembled a total of 4,932 samples from 1000 Genomes Project (The 1000 Genomes Project Consortium et al., 2010; The 1000 Genomes Project Consortium et al., 2012) and the African Genome Variation Project (AGVP) (Gurdasani et al., 2015). We have detailed the integration of these data in our previous work (Chimusa et al., 2022). Based on the initial sample description (population or country labels), we used the ethnolinguistic information (Gudykunst and Schmidt, 1987; Michalopoulos, 2012) to categorize the obtained data per ethnic group and define 20 global ethnolinguistic groups as described in Supplementary Table S2. The populations are African-American, African-Caribbean, Afro-Asiatic, Afro-Asiatic Cushitic, Afro-Asiatic Omotic, Afro-Asiatic Semitic, Latin American, Khoe-San, Niger-Congo Bantu Center, Niger-Congo Bantu South, Niger-Congo Volta, Niger-Congo West, European North, European South, United States European, European Center, East Asian, South Asian, United Kingdom South Asian and United States Indian.

#### Assessment of population structure and admixture

We merged the 4,932 samples from 1000 Genomes Project and AGVP with our 390 Botswana samples resulting in a final total of 5,322 samples using PLINK (Purcell et al., 2007) as per our previous approach (Choudhury et al., 2017; Choudhury et al., 2020). We merged datasets based on common SNPs from autosomal chromosomes using the most-parsimonious alleles from the human genome reference (GRCh38), carried out quality control and pruning of the merged dataset (Choudhury et al., 2017; Choudhury et al., 2020; Chimusa et al., 2022). Unaligned alleles were solved by strand flipping with 1000 Genomes alleles as a reference. Variants were pruned to remove those with minor allele frequency <5%, >2% missingness, those that deviated from Hardy-Weinberg Equilibrium (HWE *p* > 1.0 × 10^−5^), and those in linkage disequilibrium (LD) *r* ^2^ > 0.85 within 1000 kb window size, incrementing with 50 bases step (--indep-pairwise 1000 50 0.15). Pairwise allele sharing (identity-by-descent, IBD) was determined using pi_hat threshold of 0.2 (--genome --min 0.2). This resulted in 258,773 variants retained for assessing population diversity.

To assess structure between the population of Botswana and the 20 ethnolinguistic populations, PCA implemented in the EIGENSTRAT/smartpca programme of the EIGENSOFT package (Patterson et al., 2006; Price et al., 2006) was applied to the merged dataset. We also evaluated the extent of substructure within the Botswana population. Population structure and admixture were visualized by PCA plots generated using Genesis software (Buchmann and Hazelhurst, 2015) and R (R Core Team, 2022). The ADMIXTURE (Alexander et al., 2009) algorithm was used to estimate the ancestry proportions of the Botswana HIV-positive and -negative groups. The accurate admixture cluster was identified from model inference with lowest cross-validation (CV) error and the genome-wide admixture proportion estimations of that model inference were used as accurate genetic ancestry contribution (Supplementary Figure S2). From these, and also basing on the population history of Southern Africa (Thami and Chimusa, 2019), we chose the best 3 proxy ancestral populations that had the highest genome-wide ancestry proportions from admixture analysis: Niger-Congo, Khoe-San and European.

#### Genetic distance (F_ST_) and inbreeding analysis

Pairwise genetic distance was estimated between the Botswana population and the 20 global ethnic populations using the Weir and Cockerham’s F_ST_ (Weir and Cockerham, 1984) in PLINK. A heatmap and hierarchical clustering of the genetic distances was generated using the ComplexHeatmap package (Gu et al., 2016) in R (R Core Team, 2022). We used PLINK to calculate homozygosity by keeping some of the default parameters while adjusting the window length and number of heterozygous SNVs allowed in the window (--homozyg-kb 150 and --homozyg-window-het 3). We visualized and compared the median lengths and segments of the runs of homozygosity (ROH) between the Botswana individuals and other world ethnic groups using Mann-Whitney U test using R (R Core Team, 2022) and ggplot2 (Wickham, 2016).

#### Variants annotation and mutation prioritization

Gene-based annotation for each population VCF file to determine whether the variants putatively cause protein coding changes was performed using ANNOVAR (Wang et al., 2010), with loss-of-function validations done through snpEFF version 4.3T (Cingolani et al., 2012). We used ANNOVAR “2016Dec18” setting, where the population frequency, pathogenicity for each variant was obtained from 1000 Genomes exome (The 1000 Genomes Project Consortium et al., 2015), Exome Aggregation Consortium (Karczewski et al., 2017) (ExAC), targeted exon datasets and COSMIC (Forbes et al., 2015). Gene functions were obtained from RefGene (O’Leary et al., 2016) and different functional predictions were obtained from ANNOVAR’s library. A total of 14 predictions that included 7 functional prediction scores (SIFT (Ng and Henikoff, 2003; Sim et al., 2012), LRT (Chun and Fay, 2009), MutationTaster (Schwarz et al., 2014), MutationAssessor (Reva et al., 2011), FATHMM (Shihab et al., 2013; Shihab et al., 2014), Polyphen2 HVAR (Adzhubei et al., 2010), Polyphen2 HDIV (Adzhubei et al., 2010)), 3 ensemble scores (RadialSVM (Kircher et al., 2014), LR (Kircher et al., 2014), CADD (Kircher et al., 2014; Rentzsch et al., 2019)), and 4 conservation scores (GERP++ (Cooper et al., 2005), PhyloP-placental (Garber et al., 2009), PhyloP-vertebrate (Garber et al., 2009) and SiPhy (Garber et al., 2009)). From each resulting functional annotated dataset, we independently filtered for predicted functional status (of which each predicted functional status is of “deleterious” (D), “probably damaging” (D), “disease_causing_automatic” (A) or “disease_causing” (D)) from SIFT, LRT, MutationTaster, MutationAssessor, FATHMM, RadialSVM, LR, CADD, GERP++, Polyphen2 HVAR, Polyphen2 HDIV, PhyloP-placental, PhyloP-vertebrate and SiPhy.

As for our previous work (Chimusa et al., 2022), we prioritized the variants by retaining a variant only if it had at least 10 predicted functional status “D” or “A” out of 14 (Wonkam et al., 2020; Chimusa et al., 2022). We classified the most deleterious variants as those that were assigned “D” by FATHMM (Shihab et al., 2013; Dong et al., 2014; Shihab et al., 2014), a disease-specific weighting scheme, which uses a Hidden Markov Models prediction algorithm capable of discriminating between disease-causing mutations and neutral polymorphisms. FATHMM has been found to have the most discriminative power among other individual *in silico* mutation prediction tools (Dong et al., 2014). We identified additional deleterious variants within the prioritized genes with snpEFF loss-of-function (LOF) module (Cingolani et al., 2012).

#### Distribution of minor allele frequency and gene-specific in SNP frequencies

The distribution of the minor allele frequency of variants within HIV-1 specific host genes across the 20 global populations (Supplementary Table S2) was investigated. To this end, the proportion of minor alleles were categorized into 6 bins (0-0.05, 0.05-0.1, 0.1-0.2, 0.2-0.3, 0.3-0.4, 0.4-0.5) with respect to each group. The minor allele frequency (MAF) per SNP for each category was computed using PLINK software (Purcell et al., 2007). Furthermore, the aggregated SNP frequency in each gene was computed considering SNPs upstream and downstream of the gene region that are in close proximity and possibly in LD (Chimusa et al., 2016; Chimusa et al., 2022). We obtained a list of 730 HIV associated genes from GWAS Catalog (www.ebi.ac.uk/gwas/), literature and gene-diseases database such DisGeNET (disgenet.org). We also leveraged the dbSNP151 database (https://www.ncbi.nlm.nih.gov/snp/ (Sherry et al., 2001)) to extract SNVs associated with these genes in the Botswana dataset (Supplementary Table S3).

#### Pathways enrichment analysis and gene-gene interactions

The GeneMANIA (Warde-Farley et al., 2010) tool was used to analyse how the genes harbouring the most deleterious variants (in the Botswana population) interact in a biological network. This allowed us to obtain an enrichment of related genes within the obtained sub-network with potentially affected biological pathways, processes, and molecular functions. Gene-set enrichment analysis was performed using Enrichr package (Chen et al., 2013; Kuleshov et al., 2016) in R (R Core Team, 2022).

## Results

### Assessment of population structure and admixture

**FIGURE 1:**
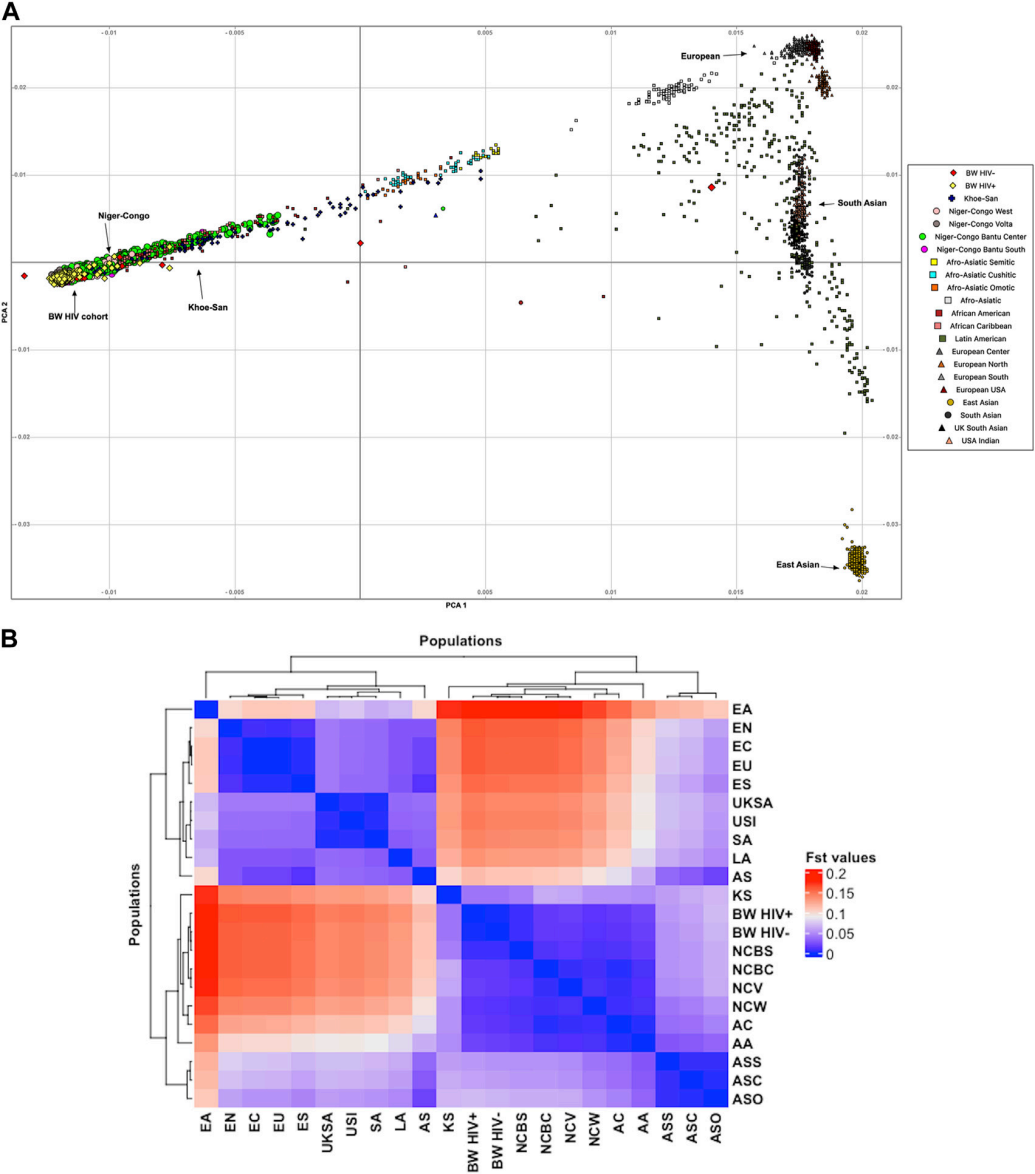
Botswana population structure in relation to the global population structure. **(A)** PCA showing genetic relationship between the Botswana and global populations. **(B)** Pairwise genetic distance between the Botswana population and 20 global ethnic groups. This is a heatmap and dendrogram of F_ST_ values showing pairwise genetic divergence between populations. The blue shade represents similarity while the red shade represents divergence between the populations. The populations are AA, African-American; AC, African-Caribbean; AS, Afro-Asiatic; ASC, Afro-Asiatic Cushitic; ASO, Afro-Asiatic Omotic; ASS, Afro-Asiatic Semitic; LA, Latin American; KS, Khoe-San; BW HIV+, Botswana HIV-1 positive; BW HIV-, Botswana HIV-1 negative; NCBC, Niger-Congo Bantu Center; NCBS, Niger-Congo Bantu South; NCV, Niger-Congo Volta; NCW, Niger-Congo West; EN, European North; ES, European South; EU, United States European; EC, European center; EA, East Asian; SA, South Asian; UKSA, United Kingdom South Asian and USI, United States Indian.

Population structure was assessed within the population of Botswana, and between the Botswana population and other global populations using 258,773 shared bi-allelic variants. The Botswana population formed a cluster with other African populations of the Niger-Congo ethnolinguistic phylum, away from the other ethnicities (Figure 1A). In addition, the results from the pairwise genetic distance (F_ST_) (Figure 1B) accentuates what was observed in assessment of global population structure with PCA. The heatmap and hierarchical clustering shows two distinct clusters separating into the Eurasian and African clades. A sub-clade that branches into the Niger-Congo populations and the Khoe-San population was observed. An inner sub-clade that separates Southern Bantu-speakers (including the Botswana population) from other Niger-Congo population is also observed. We also assessed the genetic relationship between the Botswana population, other Niger-Congo populations and the Khoe-San. We see in Figure 1B that the Botswana population and the Niger-Congo Bantu South (Zulu people of South Africa) formed a separate cluster from other Niger-Congo populations. The Botswana population showed a closer affinity with the Niger-Congo Bantu South population. This is expected as a close affinity of the Sotho with the Niger-Congo Bantu South has previously been reported (Choudhury et al., 2017) and our sampling sites are populated with Setswana speaking ethnic groups. These groups are members of the Sotho-Tswana clan of Southern Africa that includes the Sotho (of Lesotho and South Africa) and Batswana (of Botswana and South Africa) (Batibo, 1999; Berman, 2017). Within population substructure was not observed in the Botswana population. The plot of the first 2 PCs show a homogeneous mix of individuals from the HIV positive and the HIV negative groups with 3 outliers (Figure 3A).

**FIGURE 2:**
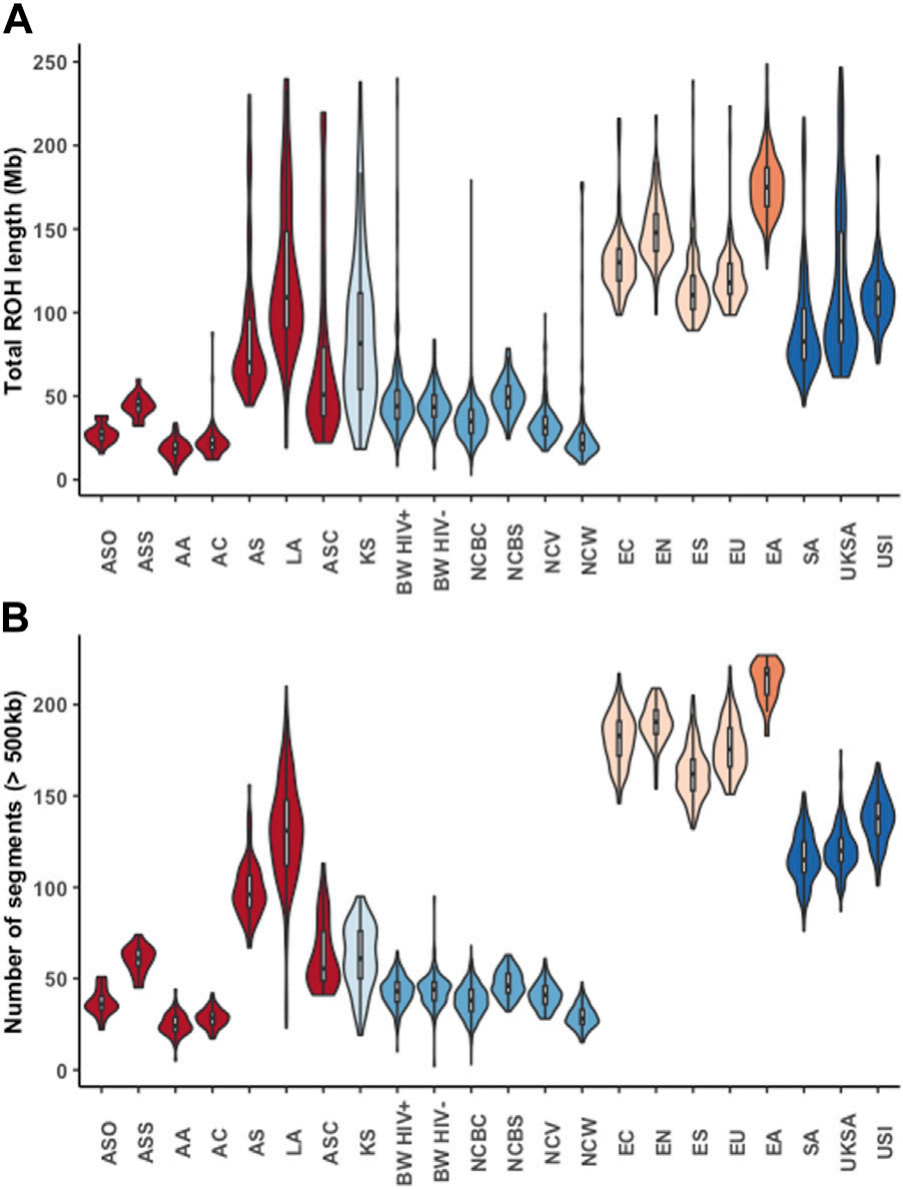
The lengths and number of runs of homozygosity (ROH) segments across different global ethnic groups. Violin plots showing the median lengths (in Mb) and number of ROH segments. The colours represent different super-groups: Mixed populations (African-American (AA), African-Caribbean (AC), Afro-Asiatic (AS), Afro-Asiatic Cushitic (ASC), Afro-Asiatic Omotic (ASO), Afro-Asiatic Semitic (ASS) and Latin American (LA)) in dark-red, Khoe-San (KS) in light blue, Niger-Congo (Botswana HIV-1 positive (BW HIV+), Botswana HIV-1 negative (BW HIV-), Niger-Congo Bantu Center (NCBC), Niger-Congo Bantu South (NCBS), Niger-Congo Volta (NCV) and Niger-Congo West (NCW)) in blue, Europeans [European North (EN), European South (ES), United States European (EU), European center (EC)] in light orange, East Asian (EA) in orange and South Asians [South Asian, (SA), United Kingdom South Asian (UKSA) and United States Indian (USI)] in orange.

Furthermore, our results showed diversity in the runs of homozygosity (ROH) segments among African populations, and between the African populations and non-African populations (Figure 2; Supplementary Table S4). Generally, the Niger-Congo populations (including the Botswana cohort) had lower ROH lengths and less abundant ROH segments than the European, Asian, Indian, Latin-American and Khoe-San populations (Figure 2). For instance, we observe a lower median ROH length (*p*-value <2.2 × 10^−16^) in Niger-Congo (35.446 Mb; IQR: 27.704, 43.267) populations than in the European populations (121.306 Mb; IQR: 109.443, 138.028).

**FIGURE 3:**
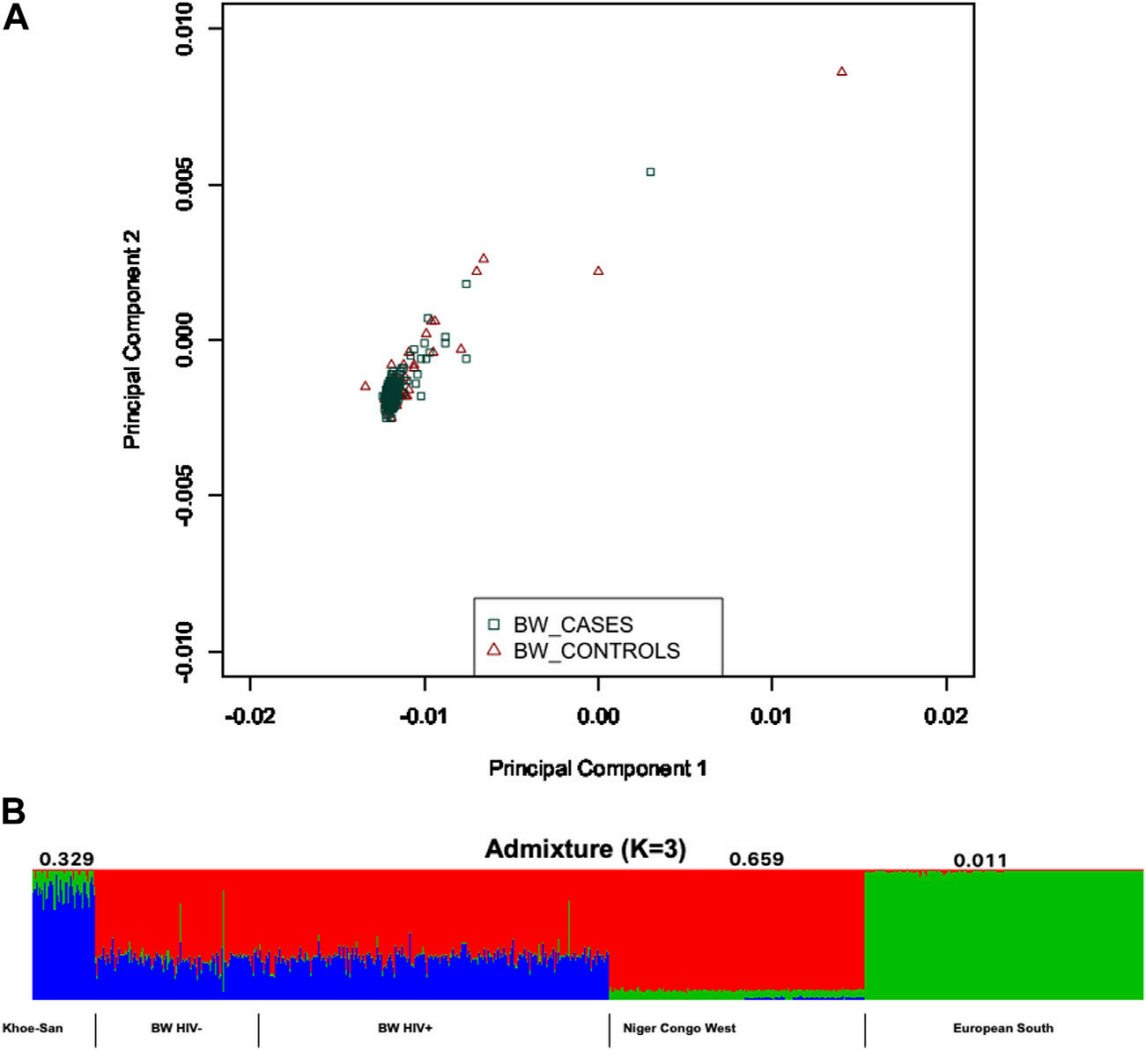
Within population diversity of the Botswana population. **(A)** A depiction of population substructure of Botswana from PCA showing genetic relationship between HIV positive in green and HIV negative in brown. **(B)** Genome-wide admixture proportions of Botswana. Khoe-San, Niger-Congo and European populations were used as proxy ancestral populations that may have potentially contributed to the genetic architecture of Botswana.

Given the results in Figure 1A, we performed admixture analysis to estimate the individual fraction of genetic ancestry. The optimal admixture model (see Materials and Methods) was the one that showed stability (K = 3) in estimation of ancestry proportions and with the lowest cross-validation (CV) value (Figure 3B). This estimated number *K* = 3 is consistent with the number of source populations from literature that contributed to the admixture of East and Southern African populations (Tishkoff et al., 2009; Pickrell et al., 2012; Chimusa et al., 2013a; Pickrell et al., 2014; Gurdasani et al., 2015; Busby et al., 2016; Choudhury et al., 2017; Retshabile et al., 2018; Choudhury et al., 2020). The Botswana population assessed in this study shows elevated components shared with the following ancestry proportions: Niger-Congo (65.9%), Khoe-San (32.9%) and Eurasian (1.1%) (Figure 3B).

### Characterization of variants and variants effect in the Botswana population

**FIGURE 4:**
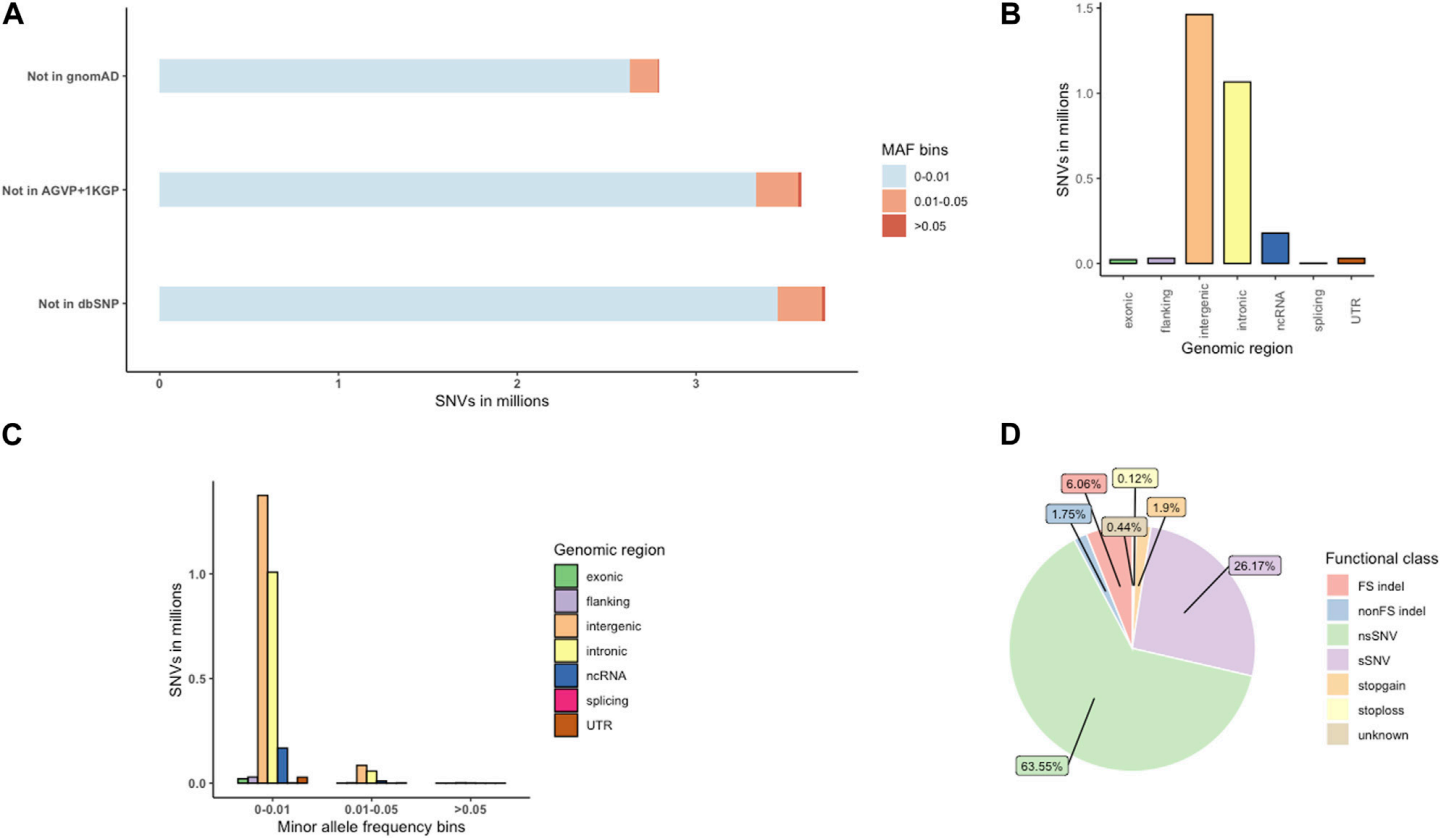
The distribution of novel variants in the Botswana population genomes. **(A)** Novel variants, absent from dbSNP151, the African Genome Variation Project (AGVP), the 1000 Genomes Project (1KGP) and gnomAD. **(B)** Genome-wide distribution of novel variant effects by functional elements. **(C)** Distribution of novel functional elements across MAF bins **(D)** Distribution of exonic variants by functional elements. FS, frameshift; sSNV (synonymous SNVs); nsSNV (non-synonymous SNVs).

We provide a broad survey of polymorphisms in whole genome sequences of 390 unrelated HIV positive and negative individuals from Botswana. A total of 265 HIV-1 positive and 125 HIV-1 negative individuals passed WGS analysis quality control. The demographics of the study population are presented in Supplementary Table S1A (Materials and Methods). We identified 27.7 million variants from the 390 individuals of Botswana. Of these 27.7 million variations, we found 25.1 million SNVs and 2.6 million indels (Supplementary Table S1B); 2,789,599 (10.08%) of these variations were novel, i.e., not found in dbSNP151, 1000 Genomes Project (1KGP), African Genome Variation Project (AGVP) and Genome Aggregation Database (gnomAD) (Karczewski et al., 2020) (Figure 4A). The average transition-transversion (Ti/Tv) ratio was 2.1, which is within the expected Ti/Tv ratio for whole genomes.

The novel variants were classified into which genomic position the variants occur, and what consequence they have on the transcript or encode gene. Positional annotations show whether the variant overlaps the following regions: coding (exonic), intron (intronic), intergenic region, 1-kb region upstream or downstream of transcription start site (upstream, downstream), a transcript without coding annotation (ncRNA), 5′untranslated region or 3′untranslated regions (5′UTR,3′UTR). Exonic variants are further classified into functional consequences: synonymous (does not cause an amino-acid change), nonsynonymous (causes an amino-acid change), frameshift (changes the open reading frame of the coding sequence), stopgain (introduces a stop codon at the variant site), stoploss (removes a stop codon at the variant site) and variants of unknown function (Wang et al., 2010; Wang, 2023).

Intergenic variants were observed at the highest frequency (1,461,193; 5.28%), followed by intronic (1,066,166; 3.85%) and ncRNA (178,178; 0.64%) variants (Figure 4B; Supplementary Table S1B). Most of the novel (2,786,546; 99.89%) variants were singletons, rare (MAF <0.01) and low frequency variants (MAF 0.01-0.05) (Supplementary Table S5; Figures 4A, C). Potentially protein altering variants (nonsynonymous SNVs (nsSNV), stop gain, stop loss variants, frameshift (FS indel) and non-frameshift (nonFS) indels), synonymous (sSNV) and variants of unknown consequence formed 73.39% (15,899), 26.17% (5,670) and 0.44% (Chan et al., 2019) of the exonic variants respectively (Figure 4D; Supplementary Table S1B).

### Variant prioritization and prediction of mutation burden

Potentially pathogenic SNVs were identified by selecting variant predictions of deleteriousness (Supplementary Table S6) from at least 10 out of 14 predictive tools using ANNOVAR (Wang et al., 2010) (Materials and Methods). We identified deleterious variants in a list of 24 genes that are all known HIV-1 specific genes (*TTLL10*, *ACTRT2*, *ENO1*, *CYP4A22*, *PM20D1*, *HOXD12*, *DNAH7*, *PDHA2*, *LRBA*, *DCHS2*, *VCAN*, *ADGRV1*, *MRPS18A*, *ABCB5*, *AKR1B10*, *ADAM7*, *OR51A4*, *OR2D2*, *KRT76*, *OR6S1*, *ATP8B4*, *ABCC12*, *OR3A1*, *PHF20*) (Supplementary Table S6). We trimmed this list of these genes by further classifying variants as “damaging” by FATHHM (Shihab et al., 2013; Shihab et al., 2014). This resulted in 5 most deleterious mutations within the *ACTRT2*, *HOXD12*, *ABCB5*, *ATP8B4* and *ABCC12* genes (Table 1).

**TABLE 1:** The most deleterious nonsynonymous single nucleotide variants.

CHR	Position	ID	AA change	Gene	Botswana	1KGP	gnomAD__AFR_
1	3022425	rs3795263	p.G247R	*ACTRT2*	A = 0.0013	A = 0.12	A = 0.044
2	176100737	rs200302685	p.E264Q	*HOXD12*	C = 0.032	—	C = 0.00038
7	20727068	rs111647033	p.R440P	*ABCB5*	C = 0.026	C = 0.0004	C = 0.0022
15	49972713	rs77004004	p.P371H	*ATP8B4*	T = 0.019	T = 0.0038	T = 0.013
16	48139198	rs113496237	p.G266R	*ABCC12*	G = 0.013	—	G = 0.000071

CHR, chromosome; AA change, amino acid change; 1KGP, The 1000 Genomes Project MAF; gnomAD__AFR_, MAF of an African population from the gnomAD database.

### Distribution of MAF in known HIV-1 specific host genes

**FIGURE 5:**
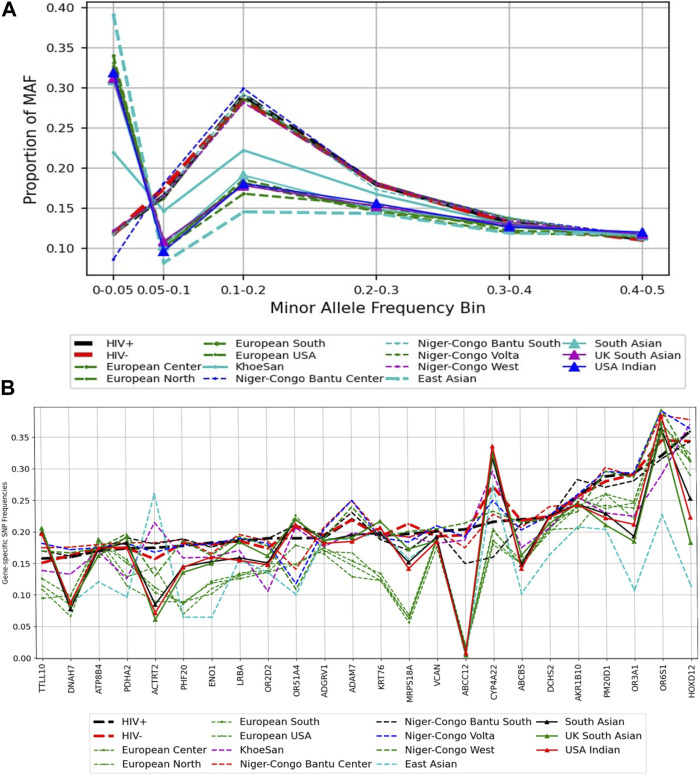
A comparison of minor alleles frequencies across global populations from known HIV-1 associated host genes. **(A)** Distribution of variants across MAF bins in global populations. **(B)** Gene-specific SNPs Frequencies: the distribution of the minor allele frequency at the gene level.

We used variants extracted from the prioritized list of the 24 genes that harboured deleterious variants. We observed variation in the distribution of MAF at rare and common variants between Botswana HIV-positive and -negative group, and the rest of 20 ethnolinguistic groups, except Niger-Congo Bantu that has similar pattern of the distribution of MAF with Botswana HIV -positive and -negative group (Figure 5A). Botswana HIV-1 cohort and Niger-Congo populations have low proportion of rare and low frequency variants (between 0% and 5%) and relatively high proportion of common variants (greater than 5%–30%). This is not surprising as African population have the highest genetic diversity. The results might imply that the multiple common variants affect genetic predisposition or resistance to HIV-1 among Africans (Thami and Chimusa, 2019). We observe variation in the aggregated SNP frequency in known HIV-1 specific host genes between African ethnolinguistic groups and those out of Africa (See Materials and Methods). Importantly, variation in the aggregated SNP frequency is observed in the *CYP4A22, AKR1B10, HOXD12, OR6S1, MRPS18A, ORS1A4 and ACTRT2* genes (Figure 5B; Supplementary Table S8) between Botswana HIV-positive and -negative population, suggesting that these genes may harbour differing effects among Botswana HIV-positive and -negative population.

### Pathways enrichment analysis and gene-gene interactions in the Botswana population

**FIGURE 6:**
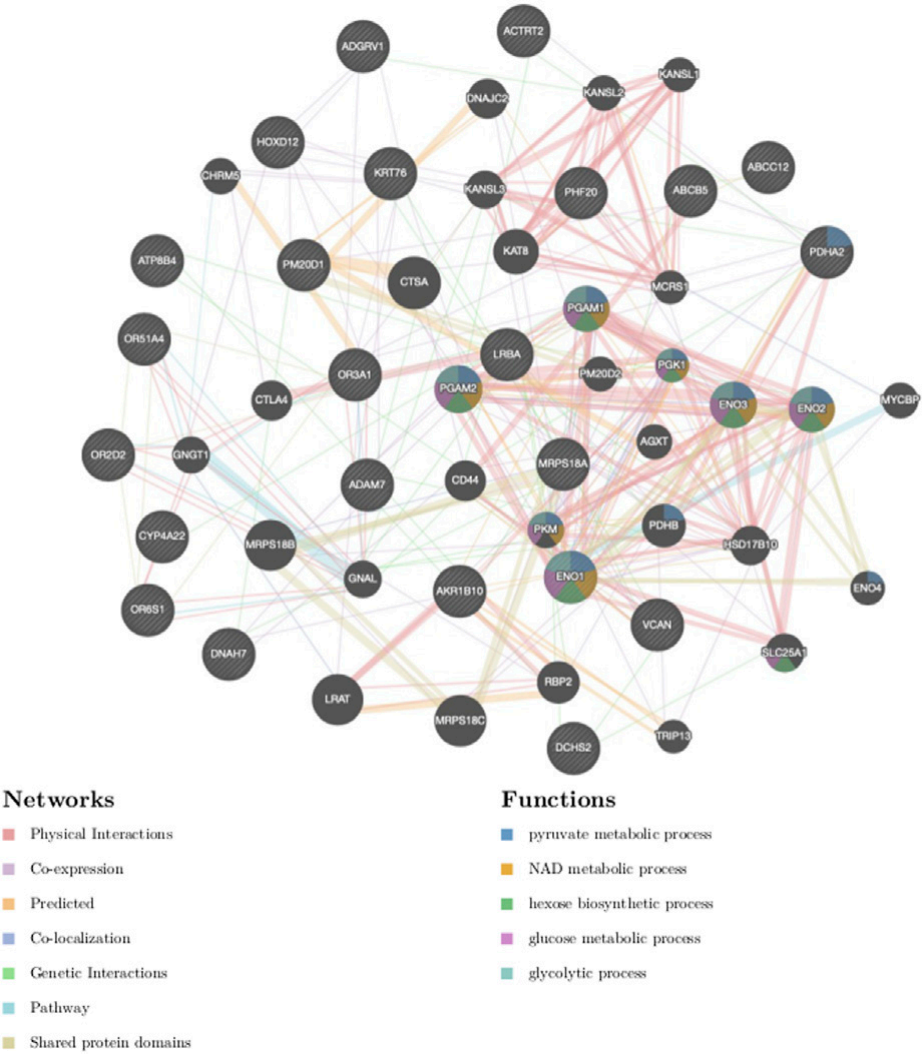
Gene-gene interaction network of genes harbouring the most deleterious variants. The different colours of branches represent how the genes are related; pink: physical interactions, purple: co-expression, orange: predicted, navy blue: co-localization, blue: pathway, green: genetic interactions, yellow: shared protein domains. Black and stripped nodes: genes provided as input into GeneMANIA. Black only nodes: genes predicted by GeneMANIA to interact with the input list. Connecting lines represent interactions.

The 24 genes (Table 1; Supplementary Tables S6, S7) harbouring potentially pathogenic variants were subjected to enrichment analysis using GeneMANIA (Warde-Farley et al., 2010) and Enrichr (Kuleshov et al., 2016) bioinformatics tools to identify biological processes and pathways putatively perturbed (Figure 3; Table 2). To successfully enrich for biological processes and pathways, the identified genes were used to find 20 more related genes that are co-expressed and predicted to physically interact with the identified genes (Figure 6).

**TABLE 2:** Enrichr gene-set enrichment of the genes harbouring the prioritized mutations.

Name	*p*-value	P-value_adj_	Database
Gene ontology			
Hexose biosynthetic process	2.59e^−6^	1.32e^−2^	Biological Process 2018 (Harris et al., 2004) http://www.informatics.jax.org/
Regulation of acyl-COA biosynthetic process	2.80e^−6^	7.14e^−3^	
Pyruvate metabolic process	3.11e^−6^	3.96e^−3^	
Glucose metabolic process	1.18e^−5^	1.2e^−2^	
Gluconeogenesis	9.99e^−5^	5.1e^−2^	
Oxoglutarate dehydrogenase complex	3.46e^−5^	1.54e^−4^	Cellular Component 2018 (Harris et al., 2004) http://www.informatics.jax.org/
Mitochondrial small ribosomal subunit	2.80e^−3^	6.24e^−3^	
Mitochondrial matrix	5.57e^−5^	8.28e^−2^	
Alcohol dehydrogenase (NADP+) activity	9.62e^−8^	5.54e^−5^	Molecular Function 2018 (Harris et al., 2004) http://www.informatics.jax.org/
Exopeptidase activity	1.32e^−4^	3.80e^−2^	
Hydro-lyase activity	1.32e^−4^	3.04e^−2^	
ATPase activity, coupled to movement of substances	2.54e^−4^	4.17e^−2^	
Pathways			
Glycolysis and Gluconeogenesis	2.84e^−6^	1.34e^−3^	WikiPathways 2019 Human (Slenter et al., 2018)
Hereditary leiomyomatosis and renal cell carcinoma pathway	1.10e^−5^	2.6e^−3^	KEGG 2019 Human (Kanehisa and Goto, 2000)
HIF-1 signalling pathway	2.08e^−9^	3.20e^−7^	Panther 2016 (Mi et al., 2017)
Citrate cycle (TCA cycle)	5.57e^−9^	5.72e^−7^	
RNA degradation	2.71e^−5^	2.09e^−3^	
Central carbon metabolism in cancer	3.95e^−4^	1.52e^−2^	
Histidine metabolism	1.16e^−3^	3.98e^−2^	
Folate biosynthesis	1.49e^−3^	4.58e^−2^	
Diseases			
Pyruvate dehydrogenase complex deficiency	4.71e^−5^	8.57e^−3^	ClinVar 2019 (Landrum et al., 2013)
			OMIM Disease (McKusick, 1998)
Human immunodeficiency virus 1	6.64e^−1^	1.0e^0^	VirusMINT (Chatr-aryamontri et al., 2009)

P-value_adj_, adjusted *p*-value.

The products of the identified genes were predicted to perform the following biological processes: gluconeogenesis, hexose and acyl-COA biosynthesis (Table 2). These gene products are localized within the oxoglutarate dehydrogenase complex and the mitochondria. The predicted molecular functions of these gene products were catalysis of peptidase, hydro-lyase, alcohol dehydrogenase and ATPase activities. The identified genes were found to be associated with Pyruvate dehydrogenase complex deficiency (PDCD). One of the identified genes, tumor susceptibility 101 (*TSG101*), was also found to be associated with human immunodeficiency virus 1 (HIV-1), albeit not statistically significant (Table 2). The affected pathways (Table 2) included the *glycolysis and gluconeogenesis* (*p* < 2.84e-6)*, Citrate cycle (TCA cycle)* (*p* < 5.57e-9)*, HIF-1 signalling pathway* (*p* < 2.08e-9), *Hereditary leiomyomatosis and renal cell carcinoma pathways* (*p* < 1.10e-5). Importantly, published transcriptomic evidence (Akusjärvi et al., 2022) provides functional support for the role of the identified pathways, including the *Glycolysis, Gluconeogenesis and HIF-1 signalling* pathways, regulating susceptibility to HIV-1 infection. This suggests that some of the identified mutant genes may act together in these biological pathways to have a cumulative effect on HIV-1 expression.

## Discussion

Our study assessed genetic diversity and mutation bias in an HIV-1 cohort from Botswana using a whole-genome sequencing approach. We also studied diversity between the Botswana population and 20 global ethnolinguistic groups. The PCA plots revealed that the Botswana HIV-positive and -negative population is overall largely homogenous (Figure 1A). Although the Botswana HIV-1 positives and negatives almost completely overlap, there is a considerable spread in the data points (Figure 1A; Figure 3A). The spread is even more than that of European samples combined, this signifying a higher genetic diversity in the Botswana study population and African populations generally. The study participants were recruited from three districts in the southern part (Southern, Kweneng and South-East) of Botswana. Although the sampling sites do not span the whole of Botswana, the current study helps us to better understand genetic variation in the southern part of Botswana. Effective sampling that includes populations from different regions is needed to capture all the variation in the Botswana population and for better generalizability of findings.

Major events such as the “Bantu expansion” and Eurasian migration into Southern Africa have shaped the genetic landscape of the region. These events have led to varying degrees of admixture of the migrant groups and indigenous population (Tishkoff et al., 2009; Chimusa et al., 2013b; Petersen et al., 2013; Pickrell et al., 2014; Gurdasani et al., 2015; Choudhury et al., 2017; Montinaro et al., 2017; Thami and Chimusa, 2019). These previous findings are congruent with the current study that reports elevated components shared with Niger-Congo (65.9%), Khoe-San (32.9%) and European (1.1%) populations (Figure 3B).

We found no evidence of consanguinity in the Botswana HIV-1 cohort as defined by less abundant segments and lower lengths of ROH in comparison to non-African populations and the Khoe-San (Figure 2). This finding is supported by the previous observation of no extended ROH lengths in a Botswana HIV positive cohort (Retshabile et al., 2018). Among the Niger-Congo populations, the median ROH length in the Botswana HIV-1 cohort and the Niger-Congo Bantu South were significantly higher (*p* = 2.2e-16) than of the Niger-Congo Bantu, Niger-Congo West and the Niger-Congo Volta (Figure 2). These results are consistent with what was observed by Choudhury *et*. *al*., who observed that the Niger-Congo Bantu population of Southern Africa had the highest lengths of ROH compared to Niger-Congo populations of East, Central West and West Africa (Choudhury et al., 2017). Overall, the results of Figure 2 provide an interesting consequence and affirmation of the Out of Africa (OoA) founder event. The dispersal of a smaller group from Africa into Eurasian regions made decreased genetic variation and inbreeding likely, hence the longer runs of identical haplotypes in Eurasian populations.

It was not surprising to observe a considerable proportion of the Khoe-San ancestry as Botswana is one of the countries with the largest number of the Khoe-San. The Khoe-San are known to be the indigenous people of Southern Africa (Schlebusch et al., 2020). Moreover a recent study postulated that modern humans come from a Khoe-San woman who inhabited the prehistoric wetlands (Makgadikgadi-Okavango) in the northern part of Botswana (Chan et al., 2019). Although there is controversy around this recent finding, the Chan et al. study may support our previous hypothesis of that the Northern San originated in Botswana as evidenced by the rock paintings at Tsodilo Hills in North-western Botswana (Thami and Chimusa, 2019). Overtime the Khoe-San are expected to have mingled and interbred with the Niger-Congo people of Botswana. Hence, by showing shared genetic components with the Khoe-San, this work shows the pivotal role played by genetics in the reconstruction of population histories. A limitation of this study is that the Botswana population had no ethnolinguistic meta-data, as such ethnicity inferences cannot be drawn from this study. Nevertheless, population structure could still be assessed as the PCA is an unsupervised machine learning method and therefore can still give meaningful results.

For the analysis of Botswana samples only, we identified 27.7 million variants from 30X depth whole genomes of 390 individuals of Botswana. A critical and convenient QC metric to measure the quality and accuracy of genomic variation data is the Ti/Tv ratio (DePristo et al., 2011). The average Ti/Tv ratio of this set of variants was 2.1. This Ti/Tv ratio is within the expected range for human whole genome data which is ∼2.0–2.1, meaning that the data has a very low frequency of false positive variant sites (DePristo et al., 2011; Carson et al., 2014). As observed previously (Choudhury et al., 2017), intergenic variants had the highest frequency, followed by intronic variants and non-coding RNA (ncRNA) variants. Ten percent (2,789,599) of the discovered SNVs were novel. This number of previously uncaptured genetic variation highlights a potential of identifying population-specific variations through WGS. WGS also offers an opportunity to identify intronic variants and variants within non-coding regions. To this effect 1,066,166 intronic and 178,178 (ncRNA) novel variants were identified.

Recent human population expansion has resulted in a skewness towards excessive rare variants. This means that rare variants constitute a large part of the human genomic variations (Nagasaki et al., 2015; Keinan and Clark, 2012; Johnston et al., 2015; Hernandez et al., 2019; Epi25 Collaborative, 2019). Hence it is not surprising that 2,786,546 (99.89%) of the novel variants identified in the current study were very rare (Supplementary Table S5; Figures 4A, C). According to sequence ontology classifications, 73.39% of the exonic variants were potentially protein altering (Figure 4D; Supplementary Table S1B). Protein altering variants cause a change in the amino acid leading to a change in the protein sequence, an abnormal truncation or elongation of the protein, all leading to a change in the conformation and function of the encoded protein (Pagel et al., 2017). These nonsynonymous mutations have a potential to disturb normal biological processes and cause disease.

The product of *ACTRT2* gene may be involved cytoskeletal organization (GeneCards, 2020). The rs3795263 variant was previously identified as harmful and associated with a severe form of tick-borne encephalitis virus infection (Ignatieva et al., 2019). The *HOXD12* gene belongs to the homeobox (*HOX*) family of genes that encode transcription factors involved in regulation of embryonic development (Lappin et al., 2006; GeneCards, 2020). The exact role of *HOXD12* is unknown (GeneCards, 2020). The *HOX* genes have been implicated in maintenance and control of HIV-1 latency through epigenetic regulation (Khan et al., 2018).

The *ABCB5* gene belongs to the ATP-binding cassette (ABC) family that encodes proteins responsible for transmembrane transport of molecules including drugs such as doxorubicin (GeneCards, 2020). *ABCB5* is thought to also mediate chemoresistance of doxorubicin in malignant melanoma, (Whirl-Carrillo et al., 2012). The *ATP8B4* gene encodes an ATPase protein that is responsible for phospholipid translocation in the cell membrane (GeneCards, 2020). The *ABCC12* gene also encodes an ABC protein responsible for transmembrane transport of molecules. Some members of the *ABC* family regulate the efflux of HIV-1 antiretrovirals from intracellular compartments (Eilers et al., 2008; Salvaggio et al., 2017). Biological pathways potentially affected by the products of these putatively deleterious genes and their interactome are discussed in subsequent paragraphs.

The minor allele frequencies of the *HOXD12* rs200302685, *ABCB5* rs111647033, *ATP8B4* rs77004004 and *ABCC12* rs113496237 variants in the Botswana data were generally higher when comparing to the gnomAD and the 1000 Genomes Project data. While the MAF for the *ACTRT2* rs3795263 variant was lower than in the gnomAD and the 1000 Genomes Project data. This highlights that MAFs do vary per ethnicity which could affect the risk of disease differently between populations (Table 1).

The *pyruvate dehydrogenase* (*PDH*), *enolase* (*ENO*) and *aldo-keto reductase* (*AKR1*) genes (Figure 6; Table 2) were significantly associated with glycolysis and gluconeogenesis (1.34e-3). Both glycolysis and gluconeogenesis are glucose metabolism pathways; glycolysis is the catabolism of glucose (or glycogen) into pyruvate, while gluconeogenesis is the anabolism of pyruvate (from mainly proteins) into glucose (Yang and Brunengraber, 2000; Bonora et al., 2012). Identification of *ACTRT2, HOXD12, ABCB5, ATP8B4, ABCC12* genes (Table 1) involved in *Krebs cycle, renal carcinoma, Hypoxia-inducible factor 1 (HIF-1) signalling, RNA degradation, Histidine metabolism and folate biosynthesis* pathways (Figure 6) requires further study to explore their roles in modifying the HIV-1 phenotype.

Pyruvate produced from glycolysis is a substrate for TCA where acetyl-CoA, a precursor of cholesterol, is produced (Berg et al., 2002). Cholesterol is required for plasma membrane formation and integrity. Furthermore cholesterol is required for viral fusion to the host’s cell membrane for entry and virus release following assembly and maturation (egress) (Coomer et al., 2020). Oxidation of cholesterol to 25-hydroxycholesterol can block HIV-1 cell entry (Liu et al., 2013). In addition, HIV-1 infection upregulates glycolysis to meet the demands of viral replication and CD4^+^ T-cells with higher glycolysis rate are more susceptible to HIV-1 infection (Hegedus et al., 2014; Palmer et al., 2016; Valle-Casuso et al., 2019). Our results are timely and can contribute to the emerging field of immunometabolism in which therapy against HIV-1 infection is being evaluated through reduction of glycolysis and inhibition of cholesterol (Liu et al., 2013; Valle-Casuso et al., 2019; Taylor and Palmer, 2020; Shytaj et al., 2021).

The association of *AKR1* genes (Figure 6) with alcohol dehydrogenase (NADP+) activity and folate biosynthesis (Table 2) could be explained by that the alcohol dehydrogenases catalyse the reduction of NADP + to NADPH (Penning, 2015). This reaction also takes place within glycolysis, gluconeogenesis and pentose phosphate pathways (Berg et al., 2002; Bonora et al., 2012). Furthermore, there is also evidence of NADPH being produced from folate metabolism (Fan et al., 2014). The human polynucleotide phosphorylase (hPNPase^old-35^) is an evolutionary conserved RNA-degradation enzyme that has homologues in organisms such as *Escherichia coli* and yeast (Leszczyniecka et al., 2002; Das et al., 2011). In *E. coli* PNPase forms part of the degradosome with enolase and a helicase (Wilusz and Wilusz, 2008). This link between enolase and the evolutionary conserved PNPase may explain the association of the *ENO* genes with RNA degradation (Table 2). The degradation of HIV-1 mRNA in HIV-1 infected cells is important in suppressing HIV-1 replication (Hillebrand et al., 2019). Moreover, ENO-1 has been shown to prevent HIV-1 reverse transcription and ultimately decrease HIV-1 infectivity (Kishimoto et al., 2017), suggesting its translation into clinical practice. To the best of our knowledge, this is the largest study to use deep sequencing in efforts to delineate a complete genome map of the human population of Botswana and evaluate the burden of human genomic mutations in Botswana. To this effect we identified single nucleotide variants which could potentially disrupt the function of 24 genes, the most deleterious (damaging) variants being *ACTRT2* rs3795263, *HOXD12* rs200302685, *ABCB5* rs111647033, *ATP8B4* rs77004004 and *ABCC12* rs113496237 (Table 1). These variants had never been identified to be associated with HIV-1. The strength of the study is the use of several different but complementary analytical approaches to identify novel variants that are potential modifiers of HIV.

Rare and low-frequency variants constituted the bulk of novel variants that were identified in this study. This was made possible by the unique potential of deep sequencing that offers an opportunity to discover rare variants. This is important because unlike Mendelian conditions, complex traits are influenced by many small-effect variants from different genetic loci, a concept known as polygenicity (Visscher et al., 2012). The cumulative effect of rare variants plays an important role in the expression of complex traits such as HIV-1. Glycolysis, Gluconeogenesis and HIF-1 signaling, TCA and hexo-pentose pathways emerged to be the most affected by the putatively deleterious variants. These are critical physiological pathways responsible for energy production, amino-acid biosynthesis, immunity, and tumorigenesis among other roles.

Nonetheless, the study has some limitations. However, it should be noted that our focus is on deleterious variants and variation among HIV-1 positive and negative individuals and not on the genetic susceptibility or association studies of HIV-1 in Botswana as previously reported (Xie et al., 2017; Shevchenko et al., 2021). Also, the sample size is relatively modest and larger sample sizes would probably yield more findings. Nonetheless, the total number of whole genomes sequenced in the study represents one of the largest cohorts in Africa to date.

In summary, we reported a WGS study as part of ongoing and recent study (Shevchenko et al., 2021) on clinical phenotypes of HIV-1 in Africa. Notably, we generated a catalogue of candidate modifier genes and their associated pathways that clustered in pathophysiological pathways important in HIV-1 and with implications for therapeutic intervention. This study fills a critical gap in knowledge by using a WGS approach focusing on deleterious variants identified in HIV-1 positive and negative individuals, in contrast to most other studies that used a GWAS approach. This study thus makes significant contributions to present knowledge of the natural history and clinical heterogeneity in HIV-1 populations, with the potential for informing the design of new therapeutics.

## Data Availability

The WGS data used in this study is available through requests at the Botswana Harvard AIDS Institute Partnership, Institutional Data Access/Ethics Committee (info@bhp.org.bw, Ref. HREC 316/2019). All metadata, scripts, software information including additional resources used in the analyses and/or analysed to produce the results during the current study are all available in the GitHub project https://github.com/pthami07/botswana-hiv-host-genes.
