# Supplementary material for: Whole genome sequencing reveals population diversity and variation in HIV-1 specific host genes
Source: Front Genet. 2023 Dec 20;14:1290624. doi: 10.3389/fgene.2023.1290624 (PMC10765519; doi:10.3389/fgene.2023.1290624)
Supplement: Supplementary file 2 [file Table1.DOCX]

**Supplementary Table 1a.** Study participants’ demographics.

| **Variable** | **HIV-1 negative** | **HIV-1 positive** |
| --- | --- | --- |
| Female (n, %) | 93 (74.4) | 264 (99.6) |
| Age (median, IQR) | 38.07 (36 - 40.00) | 27.45 (23.72-31.85) |

**Supplementary Table 1b. Total variants discovered in the genomic data of Batswana.** A total of 27,662,062 variants were discovered in the whole genomes of 390 individuals from Botswana. We observed 2,789,599 variants out of the 27.7 million that were discovered.

| **Functional class** | **Number of variants** |
| --- | --- |
| 3’-UTR | 237,690 |
| 5’-UTR | 23,702 |
| 5’-UTR and 3’-UTR | 100 |
| downstream | 169,730 |
| exonic | 171,348 |
| exonic and splicing | 69 |
| intergenic | 15,116,866 |
| intronic | 10,085,022 |
| ncRNA exonic | 89,096 |
| ncRNA exonic and splicing | 20 |
| ncRNA intronic | 1,646,163 |
| ncRNA splicing | 578 |
| splicing | 2,868 |
| upstream | 114,776 |
| upstream and downstream | 4,034 |
| **Exonic variants** | |
| nsSNV | 91,994 |
| sSNV | 72,040 |
| FS indel | 2,964 |
| stopgain | 1,635 |
| nonFS indel | 1,635 |
| stoploss | 123 |
| unknown | 957 |
|  |  |
| **Total** | **27,662,062** |
|  |  |
|  |  |
| **Novel variants** | |
| UTR | 29,904 |
| flanking | 30,731 |
| exonic | 21,665 |
| intergenic | 1,461,193 |
| intronic | 1,066,166 |
| ncRNA | 178,178 |
| splicing | 1,762 |
| **Exonic variants** | |
| nsSNV | 13,769 |
| sSNV | 5,670 |
| FS indel | 1,313 |
| stopgain | 412 |
| nonFS indel | 380 |
| stoploss | 25 |
| unknown | 96 |
| Total | 2,789,599 |

nsSNV: non-synonymous single variant, sSNV: synonymous single variant, FS indel: frameshift indel, nonFS indel: non- frameshift indel.
