# Supplementary figures and images for: Whole genome sequencing reveals population diversity and variation in HIV-1 specific host genes
Source: Front Genet. 2023 Dec 20;14:1290624. doi: 10.3389/fgene.2023.1290624 (PMC10765519; doi:10.3389/fgene.2023.1290624)

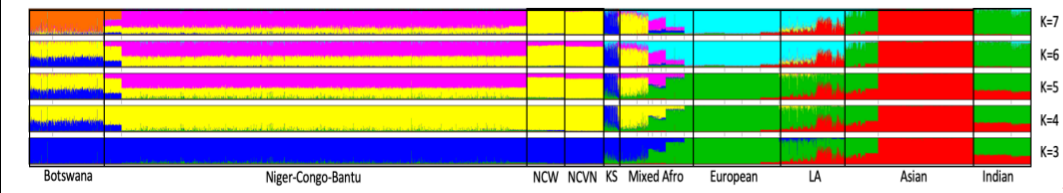

Supplement: Supplementary file 3 [file Image2.tif]

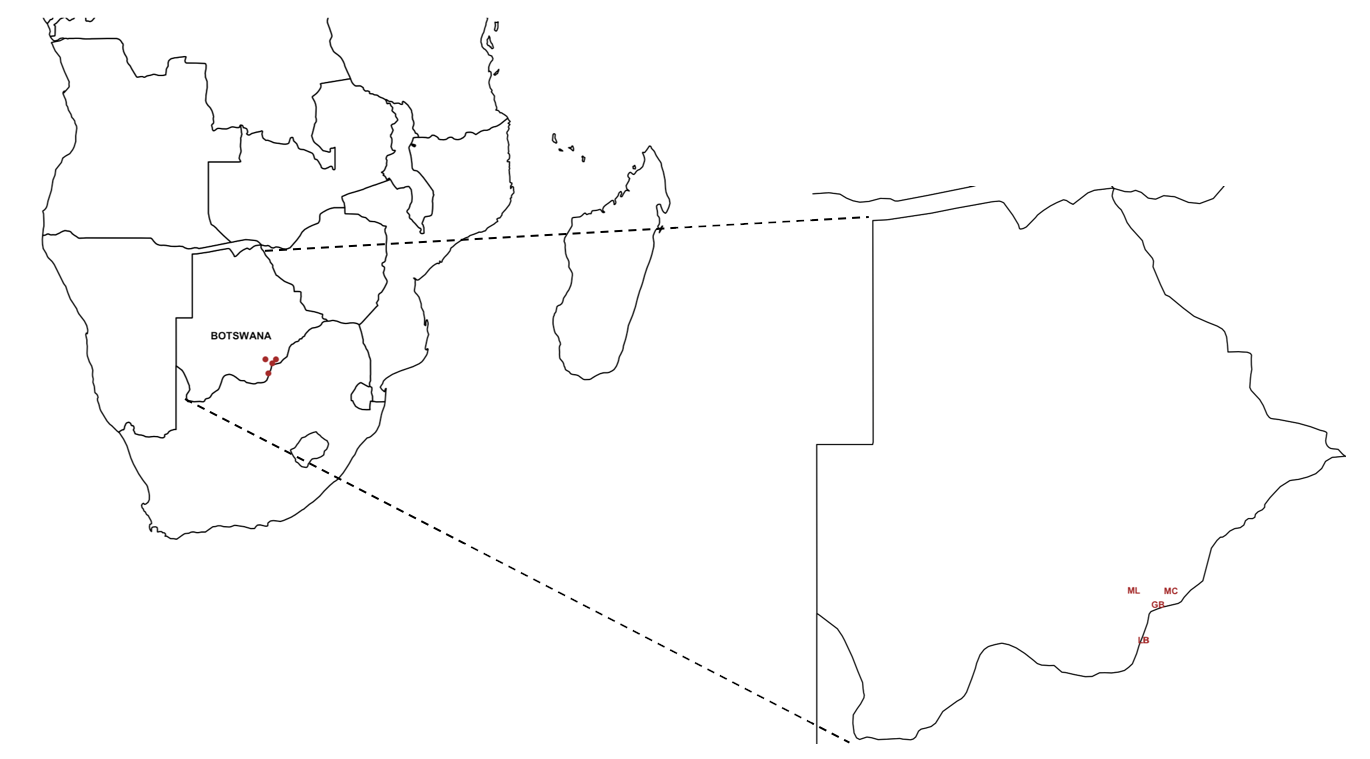

Supplement: Supplementary file 4 [file Image1.TIF]
