# Supplementary material for: Whole genome sequencing reveals population diversity and variation in HIV-1 specific host genes
Source: Front Genet. 2023 Dec 20;14:1290624. doi: 10.3389/fgene.2023.1290624 (PMC10765519; doi:10.3389/fgene.2023.1290624)
Supplement: Supplementary file 6 [file Table2.DOCX]

Supplementary Table 2. Variants data from the 1000 Genomes Project (1KGP) and the African Genome Variation Project (AGVP) used for population structure and admixture analysis.

| **Population**  **label** | **Ethnic group** | **Population description** | **Total Samples** |
| --- | --- | --- | --- |
| **AFR** | Afro Asiatic Semitic | Amhara from Ethiopia | 22 |
|  | African American | Americans of African Ancestry in SW USA (ASW) | 60 |
|  | African Caribbean | African Caribbeans in Barbado (ACB) | 96 |
|  | Afro Asiatic | Al-Gharbiyah, NA, Monufia, Kafrel-Sheikh, Mansoura, Alexandria, Dakahlia, Samanoud, Al-Buhayrah, Minya, AlSharqia, El-Mahalla all from Egypt | 99 |
|  | Afro Asiatic Cushitic | Oromo, Somali from Ethiopia | 47 |
|  | Afro Asiatic Omotic | Wolayta from Ethiopia | 24 |
|  | Khoe-San | Khoe-San | 84 |
|  | Niger-Congo Bantu Center | Baganda, Banyarwanda, Barundi, Rwandese, Ugandan, Banyankole of Uganda, Bakiga, Mutanzania, Basoga, other Uganda gwas unknown, Mutooro, Batooro, Nyanjiro (Tanzania) from Uganda and Luhya in Webuye, Kenya (LWK) | 2158 |
|  | Niger-Congo Bantu South | Zulu | 98 |
|  | Niger-Congo Volta | Esan in Nigeria (ESN), Yoruba in Ibadan, Nigeria (YRI) | 205 |
|  | Niger-Congo West | Gambian in Western Divisions in the Gambia (GWD), Mende in Sierra Leone (MSL) | 198 |
| **AMR** | Latin American | Puerto Ricans from Puerto Rico (PUR), Colombians from Medellin, Colombia (CLM), Peruvians from Lima, Peru (PEL), Mexican Ancestry from Los Angeles USA (MXL) | 347 |
| **EUR** | European Center | British of England and Scotland (GBR) | 91 |
|  | European North | Finnish of Finland (FIN) | 99 |
|  | European South | Iberian Population in Spain (IBS), Toscani of Italia (TSI) | 214 |
|  | European USA | Utah Residents with Northern and Western European Ancestry (CEU) | 99 |
| **EAS** | East Asian | Southern Han Chinese (CHS), Chinese Dai in Xishuangbanna, China (CDX), Kinh in Ho Chi Minh City, Vietnam (KHV), Han Chinese in Beijing, China (CHB), Japanese in Tokyo, Japan (JPT) | 504 |
|  | South Asian | Punjabi from Lahore, Pakistan (PJL), Bengali from Bangladesh (BEB) | 180 |
| **SAS** | UK Indian | Sri Lankan Tamil from the UK (STU), Indian Telugu from the UK (ITU) | 204 |
|  | USA Indian | Gujarati Indian from Houston, Texas (GIH) | 103 |
| **Total** | | | 4,932 |
