# Supplementary material for: Whole genome sequencing reveals population diversity and variation in HIV-1 specific host genes
Source: Front Genet. 2023 Dec 20;14:1290624. doi: 10.3389/fgene.2023.1290624 (PMC10765519; doi:10.3389/fgene.2023.1290624)
Supplement: Supplementary file 7 [file Table5.DOCX]

**Supplementary Table 5. Novel variants per MAF category.** The distribution of novel variants was compared among four classes of minor allele counts (MAC).

| **Functional class** | **MAF** | | | |
| --- | --- | --- | --- | --- |
|  | 0-0.01 | 0.01-0.05 | >0.05 | Total |
| UTR | 28370 | 1503 | 31 | 29904 |
| flanking | 28962 | 1725 | 44 | 30731 |
| exonic | 21160 | 500 | 5 | 21665 |
| intergenic | 1374111 | 85337 | 1745 | 1461193 |
| intronic | 1007198 | 57925 | 1043 | 1066166 |
| ncRNA | 167663 | 10331 | 184 | 178178 |
| splicing | 1758 | 3 | 1 | 1762 |
| Total | 2,629,222 | 157,324 | 3,053 | 2,789,599 |
