# Supplementary material for: Whole genome sequencing reveals population diversity and variation in HIV-1 specific host genes
Source: Front Genet. 2023 Dec 20;14:1290624. doi: 10.3389/fgene.2023.1290624 (PMC10765519; doi:10.3389/fgene.2023.1290624)
Supplement: Supplementary file 11 [file DataSheet1.docx]

Supplementary Material

***Materials and Methods***

***Variant Calling and Downstream Data Description***

Quality assessment was performed on paired-end WGS (minimum of 30X depth) in FASTQ format (1) using FastQC (2). Low-quality sequence bases and adapters were trimmed using Trimmomatic with default parameters (3). The sequencing reads were aligned to the GRCh38 human reference genome using Burrows-Wheeler Aligner (BWA-MEM) (4,5) and post-alignment quality control including adding of read groups, marking duplicates, fix mating and recalibration of base quality scores was performed using Picard tools, SAMtools (6) and Genome Analysis Toolkit (7). Four samples (HIV-1 positive females) were excluded due to poor quality of sequences, the remaining dataset had 390 individuals. We have run FastQC on all final BAM files prior the variant calling, then we aggregated the results from FastQC into a single report by using MultiQC (8).

Variant calling is a process of determining nucleotide differences between the reference sequence and the sequence of a sample. In population genetics, it is best to perform the identification of variants from different individuals simultaneously – a process known as population joint calling (9,10). We performed variant calling using two different population joint calling methods to leverage the quality and accuracy of our results: GATK’s HaplotypeCaller (7,11) and BCFtools (6). The variant call format (VCF) dataset was filtered using VCFTOOLS (12), GATK’s Variant Quality Score Recalibration and BCFtools. The specific filtering parameters employed for both call-sets have been detailed below. Downstream analyses were performed with GATK call-set and BCFtools call-set used as a validation set.

***Variant Calling parameters applied to the sequence data***

All the sequences passed quality control. Over 90% of the samples had a high sequence quality (at least 30 Phred score). This means that for these sequences a 99.9% accuracy in base calling was achieved. The acceptable Phred score for sequencing downstream analyses (population structure and genetic association) is at least 20. Although the sequence quality of the study samples was good, for variant calling bases with a Phred score of at least 30 were considered to ensure accurate identification of variants.

For **GATK** variant calling and filtration, we used GATK version 4.1.4.1. We performed variant calling using GATK’s HaplotypeCaller to identify potential variants in each sample, then performed population joint genotyping to ensure high accuracy of calling. The variant calling followed GATK’s best practices the following parameters:

gatk --java-options " -Xmx8g " HaplotypeCaller \

-R hg38.fasta \

-I SAMPLE.bam \

--dbsnp dbsnp151.vcf.gz \

--emit-ref-confidence GVCF \

-stand-call-conf 30 \

-O SAMPLE.g.vcf

The genotype VCF files were combined into a cohort file then population joint-calling was performed using the following parameters:

gatk --java-options " -Xmx100g" GenotypeGVCFs \

-R hg38.fasta \

--dbsnp dbsnp151.vcf.gz \

-V combine.vcf.gz \

-stand-call-conf 30.0 \

-A Coverage -A FisherStrand -A StrandOddsRatio -A MappingQualityRankSumTest \

-A QualByDepth -A RMSMappingQuality -A ReadPosRankSumTest \

--allow-old-rms-mapping-quality-annotation-data \

-O gatk.cohort.vcf.gz

For **BCFTOOLS** variant calling we used multiallelic calling model with the following parameters:

bcftools mpileup -Q30 -Ou -f hg38.fasta \

SAMPLE1.bam \

SAMPLE2.bam \

SAMPLE390.bam | bcftools call -mv -Oz -o bcf.cohort.vcf.gz

Raw variants called from GATK were filtered on minimum depth (DP) of 10, minimum genotype quality (GQ) of 20 and genotype call rate of 90% using VCFTOOLS. Following GATK guidelines, we filtered variants that had excess heterozygosity (> 54.69) and further filtered the data using machine learning framework implemented in GATK’s Variant Quality Score Recalibration (VQSR) with the above-mentioned annotation features and the following training data sets:

**INDELs**

Mills_and_1000G_gold_standard.indels.hg38.vcf (prior=12.0)

dbsnp151.vcf (prior=2.0)

**SNVs**

hapmap_3.3.hg38.vcf (prior=15.0)

1000G_omni2.5.hg38.vcf (prior=12.0)

1000G_phase1.snps.high_confidence.hg38.vcf (prior=10.0)

dbsnp151.vcf (prior=7.0)

The training models were applied to the data using GATK’s ApplyVQSR with truth sensitivity level of 99.9%.

We used the following filters on the BCFtools call set: and the following filters: depth (DP) > 10; mapping quality (MQ) > 30; variant quality (QUAL) > 20; <10% missing genotypes; <10% heterozygosity; and filtering SNP within 3bp around a gap (--SnpGap 3).

***List and description of software and tools used***

| **Software** | **URL** | **Use** |
| --- | --- | --- |
| FastQC | <https://www.bioinformatics.babraham.ac.uk/projects/fastqc/> | Quality control of raw sequence data |
| Trimmomatic | <https://github.com/usadellab/Trimmomatic> | Trimming poor quality reads and removal of adapter sequences |
| Burrows-Wheeler Aligner | <https://sourceforge.net/projects/bio-bwa/> | Read alignment |
| Picard tools | <https://broadinstitute.github.io/picard/> | Post-alignement quality control and manipulation of NGS read data |
| SAMtools | [http://www.htslib.org](http://www.htslib.org/download/) | Interacting with and post-processing of read alignments |
| Genome Analysis Toolkit (GATK) | <https://gatk.broadinstitute.org> | Interacting with and post-processing of read alignments. Variant discovery and quality control of variants. |
| MultiQC | <https://multiqc.info> | Aggregation of FASTQC results into a single report. |
| BCFtools | [http://www.htslib.org](http://www.htslib.org/download/) | Interacting with and post-processing of read alignments. Variant discovery and quality control of variants. |
| VCFTOOLS | [https://vcftools.github.io](https://vcftools.github.io/man_latest.html) | Analysing and manipulating VCF files. |
| ANNOVAR | [https://annovar.openbioinformatics.org](https://annovar.openbioinformatics.org/en/latest/) | Annotate genetic variants. |
| PLINK | <https://zzz.bwh.harvard.edu/plink/> | Assessment of genome-wide association and population stratification. |
| GeneMANIA | <https://genemania.org/> | Prediction of gene function and construction of gene networks. |
| Enrichr | <https://cran.r-project.org/web/packages/enrichR/index.html> | Gene set enrichment analysis. |
| R | <https://cran.r-project.org> | Programming software for statistical computing and graphics. |
| ADMIXTURE | <https://dalexander.github.io/admixture/> | Estimation of genomic ancestry proportions. |
| EIGENSTRAT/**smartpca** | ﻿<http://genepath.med.harvard.edu/~reich/EIGENSTRAT.htm>  <https://github.com/DReichLab/EIG> | Run Principal Component Analysis (PCA). |
| Genesis | <http://www.bioinf.wits.ac.za/software/genesis> | Generate PCA and admixture graphs. |
| ggplot2 | <https://cran.r-project.org/package=ggplot2> | Generation of graphs. |
| ComplexHeatmap | <https://bioconductor.org/packages/ComplexHeatmap/> | Generation and annotation of heatmaps. |

**REFERENCES**

1. Cock PJA, Fields CJ, Goto N, Heuer ML, Rice PM. The Sanger FASTQ file format for sequences with quality scores, and the Solexa/Illumina FASTQ variants. Nucleic Acids Res. 2010;38(6):1767–71.

2. Van Der Auwera GA, Carneiro MO, Hartl C, Poplin R, Levy-moonshine A, Jordan T, et al. From FastQ data to high confidence varant calls: the Genome Analysis Toolkit best practices pipeline. Curr Protoc Bioinforma. 2014;11(1110).

3. Bolger AM, Lohse M, Usadel B. Trimmomatic: a flexible trimmer for Illumina sequence data. Bioinformatics. 2014;30(15):2114–20.

4. Li H, Ruan J, Durbin R, Li H, Ruan J, Durbin R. Mapping short DNA sequencing reads and calling variants using mapping quality scores Mapping short DNA sequencing reads and calling variants using mapping quality scores. 2008;1851–8.

5. Li H, Durbin R. Fast and accurate short read alignment with Burrows-Wheeler transform. Bioinformatics. 2009 Jul;25(14):1754–60.

6. Li H. A statistical framework for SNP calling, mutation discovery, association mapping and population genetical parameter estimation from sequencing data. Bioinformatics. 2011 Nov;27(21):2987–93.

7. McKenna A, Hanna M, Banks E, Sivachenko A, Cibulskis K, Kernytsky A, et al. The Genome Analysis Toolkit: A MapReduce framework for analyzing next-generation DNA sequencing data. Genome Res Sep;20(9)1297-303 doi 101101/gr107524110. 2010;Sep 20(9):1297–303.

8. Ewels P, Magnusson M, Lundin S, Käller M. MultiQC: summarize analysis results for multiple tools and samples in a single report. Bioinformatics [Internet]. 2016 Oct 1;32(19):3047–8. Available from: https://doi.org/10.1093/bioinformatics/btw354

9. Pfeifer SP. From next-generation resequencing reads to a high-quality variant data set. Heredity (Edinb). 2017;118(2):111–24.

10. Nielsen R, Paul JS, Albrechtsen A, Song YS. Genotype and SNP calling from next-generation sequencing data. Nat Rev Genet [Internet]. 2011;12(6):443–51. Available from: https://doi.org/10.1038/nrg2986

11. DePristo MA, Banks E, Poplin R, Garimella K V, Maguire JR, Hartl C, et al. A framework for variation discovery and genotyping using next-generation DNA sequencing data. Nat Genet. 2011 May;43(5):491–8.

12. Danecek P, Auton A, Abecasis G, Albers CA, Banks E, DePristo MA, et al. The variant call format and VCFtools. Bioinformatics. 2011;27(15):2156–8.
